# Efficacy and determinants of vacuum bell treatment in preschool children with pectus excavatum

**DOI:** 10.3389/fped.2022.1008437

**Published:** 2022-10-13

**Authors:** Dengke Luo, Kaisheng Cheng, Miao Yuan, Chang Xu, Taozhen He

**Affiliations:** Department of Pediatric Surgery, West China Hospital of Sichuan University, Chengdu, China

**Keywords:** funnel chest, preschool, vacuum, suction, conservative treatment

## Abstract

**Objective:**

The outcome of vacuum bell (VB) treatment for preschool patients with pectus excavatum (PE) is poorly understood. We aim to investigate the short-term treatment effect of VB with a three-dimensional scanner and assess the clinical and demographic factors that might influence treatment outcomes.

**Methods:**

We conducted a chart review study to review the records of preschool patients with PE who received VB treatment in a tertiary hospital from January 1, 2021, through January 1, 2022. Demographic data and chest wall deformity assessments were recorded at follow-up, including the anterior chest wall depths and depth ratio (DR). The demographic and clinical factors influencing treatment outcomes were tested using a logistic regression model.

**Results:**

139 patients who accepted vacuum bell treatment were included in the final study analysis, with a mean age of 4.6 years and a BMI of 14.9. Forty-three patients (30.9%) with a depth of less than 3 mm met the termination criteria and showed cosmetic results. The changes in depths (*P* < 0.001) and DR (*P* < 0.001) were statistically significant in 55 patients with three or four follow-ups. Multifactor logistic regression analysis showed that initial depth (OR 0.69, 95% CI 0.58–0.84, *P* < 0.001) and treatment period (OR 1.58, 95% CI 1.23–2.04, *P* < 0.001) were independent predictors of achieving complete correction.

**Conclusion:**

VB is an effective treatment modality in preschool patients in the short-term follow-up, which is influenced by the depth of depression and the duration of treatment. However, further prospective studies are needed to confirm these results.

## Introduction

Pectus excavatum (PE) is the most common congenital deformity of the anterior chest wall, with an incidence of approximately 1 in 400 to 1 in 1,000 live births (1), which can be exercise intolerance and negatively impact the quality of life. Although there are no standardized guidelines for the management of pectus excavatum, the treatment mainly involves surgical elevation of the sunken sternum, especially the Nuss procedure (2). Since its first introduction in 1998, the optimum timing of the Nuss procedure has been postponed from preschool age to the age before puberty to ensure that the bar is fixed in the chest wall during puberty and to reduce growth-related recurrence in patients with early surgery (3). However, at the time of surgery, some psychosocial issues, such as behavioral disorders, may have already developed at preschool age (4). Consequently, surgery for PE may not be the best choice, and there are rare treatment options for preschool patients. This may result in inadequate treatment and raise the question of the management strategies in this population (5).

Vacuum bell (VB) was introduced in 2005 and has been reported as an exciting treatment option and an alternative to surgery in selected PE patients (6–8). The ability to elevate the sternum within several minutes, even in adult patients, has been approved by intraoperative thoracoscopy and chest scans of computed tomography (CT) or magnetic resonance imaging (MRI) (9, 10). However, there are few reports focused on preschool patients. In addition, heterogeneity exists in these reports regarding the treatment outcome and the criteria of excellent correction, treatment protocol, treatment compliance, and selection criteria (11–16).

Studies of VB in PE patients have suggested that age at treatment, symmetry and initial depth may affect the treatment results (7, 12, 13). However, the results in these studies were inconsistent, and the age at initial treatment was broad, ranging from infancy to old age. Thus, we conducted this chart review study to assess the efficacy of VB treatment for preschool patients, estimate clinical and demographic factors that might influence the effectiveness of VB, and provide evidence for personalized treatment.

## Materials and methods

### Patients

In this single-center chart review study, we prospectively collected data from all outpatients diagnosed with PE who were admitted to our outpatient clinic from January 1, 2021, to January 1, 2022. All patients with initial pressures below 30 kPa and less than seven years were enrolledbecause greater negative pressure may cause pain and discomfort to patients (11). We excluded patients with incomplete follow-up data, coagulation system diseases such as coagulopathies andvasculopathy, patients with interrupted follow-up for more than six months, or unwillingness to participate. Clinical data were collected for each patient every three to six months, including symmetry, depth, depth ratio (DR), initial vacuum pressures, bell wear hours per day, frequency, age, BMI, and sex. Symmetry was defined as the deepest point of PE located at the middle line. The depth was defined as the vertical distance from the anterior chest wall's most profound point of the pectus to the connecting line between the two nipples. The DR is defined as the shortest vertical distance between the deepest external point and the line segment joining the highest points on either side of the forehead divided by that line segment (17). It was obtained from a three-dimensional (3D) scanner and had good consistency with the Haller index measured from chest CT, which was used to reflect the severity of PE. Initial vacuum pressures were recorded as the pressure elevating the sternum to normal. This study was approved by the institutional review board and patient guardians, and written informed consent was obtained (approval number 20191082).

### Vacuum bell and 3D scanner

All potential candidates for VB treatment were evaluated at the outpatient clinic with a VB. Patients were selected for VB therapy when the deepest point of PE could be elevated to a normal level with a pressure of less than 30 kPa. The VB consists of several parts: a silicon ring, a polycarbonate window, a patient-activated hand pump, and a pressure meter. The 3D scanner was used to acquire body surface data on the standing position, which was used to select a suitable VB size and record treatment outcomes. 3D print VB was used in patients with significant asymmetry PE. VB therapy is a highly personalized treatment modality. All patients were recommended to use the VB for a minimum of 30 min, twice daily or more, according to patients' willingness, and supervised by guardians. The negative pressure was created through a patient-activated hand pump to elevate the sunken sternal forward.

### Follow-up and measurement

Guardians were given a standardized diary to record daily VB usage time, frequency, pressures, and complications. Routine clinical follow-ups were conducted in all patients every three to six months with clinical and 3D scanner assessments. Patients who could not go to the outpatient clinic reported the status of daily VB use details by filing online questionnaires or answering telephone questions. The whole course of treatment was at least one year and might be extended as appropriate. At each follow-up, the data obtained from the 3D scanner were analyzed by Materialise Magics (version 21.0) to measure depth and DR. Complete correction was defined as a depth of less than 3 mm.

### Statistical analysis

The descriptive data are presented as the mean and standard deviation (means ± SDs) or median and interquartile range (IQR) for continuous variables and counts and percentages for categorical variables. The Kolmogorov–Smirnov test was used to test distribution. The Wilcoxon signed-rank test was chosen to compare pre-wearing and post-wearing depths to test the effectiveness of VB. The subgroups of special interest (daily usage times and initial pressures) were analyzed using the Mann–Whitney U test, two sample independent t-test, or chi-square tests as appropriate. Hours of VB wear per day and initial pressure were treated as binary variables for analyses (group 1: <1 h per day or <15 kPa; group 2: ≥1 h per day or ≥15 kPa). To identify the associations between demographic and clinical variables and excellent outcomes, we performed the univariable logistic analysis in which the variables with *P* < 0.2 were included in the multivariable analysis model. All reported *P* values are two-sided, and *P* < 0.05 was considered statistically significant. All analyses were performed with SPSS software (version 26.0, IBM).

## Results

### Demographics

**Figure 1:**
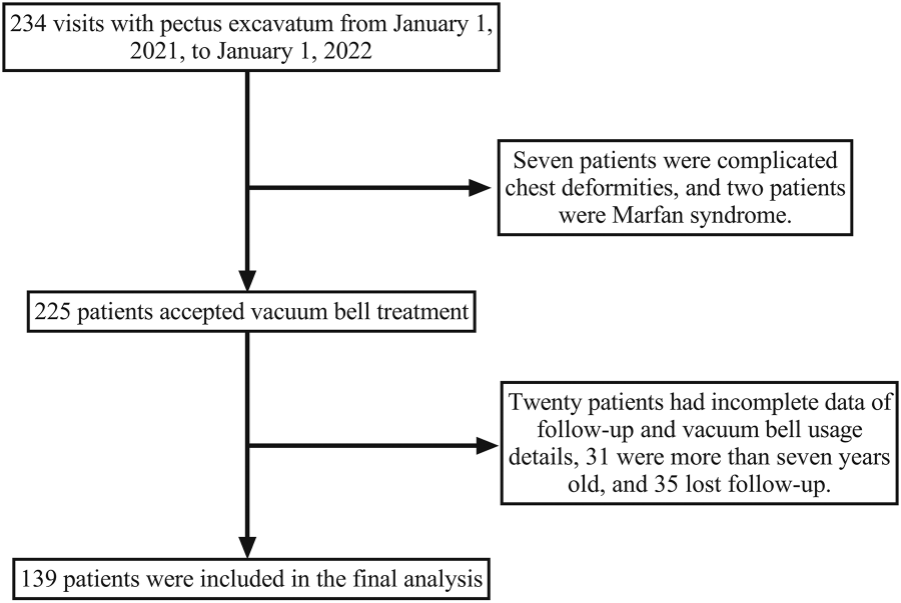
Study flow chart.

A total of 234 consecutive patients with pectus excavatum were admitted to our outpatient clinic, and findings from the final study sample of 139 patients are summarized in Figure 1. Its demographic characteristics are revealed in Table 1. Thirteen patients (9.4%) presented with comorbidities, including inguinal hernias in four cases, atrial septal defects in two cases, asthma in two cases, congenital lung malformations in two cases, Kawasaki disease in one case, glucose-6-phosphate dehydrogenase deficiency in one case, and congenital megaureter in one case.

**Table 1:** Patient demographic characteristics.

Characters	Median (IQR)/Mean	No. (%)
Age	4.6 ± 1.7	
Male		87 (63%)
Symmetry		120 (86.3%)
BMI	14.9 ± 1.4	
Comorbidity		13 (9.4%)
Symptoms		0
Family history		36 (25.9%)
Thoracic surgery history		2 (1.4%)
Initial pressure	15 (12, 20)	
Complications		40 (28.8%)

### Efficacy and safety

At the latest follow-up, forty-three patients (30.9%) with a depth of less than 3 mm met the termination criteria and required only occasional use for consolidation. Four patients discontinued VB treatment, one of whom opted for surgery. The rest of the patients continued with VB treatment.

All patients with a median of 9 (IQR 7–10) months were followed up at least once.

Of these, thirty-seven patients were followed up once (5 months, IQR 4–6), forty-seven patients were followed up twice (9 months, IQR 8–9), fifty-two patients were followed up three times (10 months, IQR 10–12), and three patients were followed up four times (12 months, IQR 12–12). The median depths and DR at each follow-up visit decreased steadily. The median depth on admission to our clinic was 9.76 (IQR 9.65–10.38) mm, while the median depth at the last follow-up was 4.05 (IQR 2.70–6.71) mm. The median depth decrease during treatment was 3.94 (IQR 2.1–6.7) mm. Similarly, the median DR on admission was 0.13 (IQR 0.10–0.18), and the median DR at the last follow-up was 0.08 (IQR 0.05–0.12). The median decrease in DR during treatment was 0.04 (IQR 0.02–0.08). The changes in depths and DR values of 55 patients with more than three follow-up visits showed statistically significant differences (Z = −6.426, *P *< 0.001 and Z = −6.292, *P* < 0.001, respectively).

Clinical outcomes of patients with different usage times or initial pressures between the two groups were compared in the stratified analysis. The two groups of different usage times demonstrated no significant differences in age (*P* = 0.431), BMI (*P* = 0.521), treatment period (Z = −0.405, *P* = 0.685), pre-wearing DR (*P* = 0.081) or depth (*P* = 0.065). When pre-wearing and post-wearing were compared, a significant reduction in depths and DR values between the two groups was demonstrated (Z = −2.957, *P* = 0.003 and Z = −2.484, *P* = 0.013, respectively). However, there was no statistical significance in the rate of acquiring excellent outcomes between the two groups (*P* = 0.928). Moreover, the two groups of different initial pressures showed no significant differences in the treatment period (Z = −0.503, *P* = 0.615) or changes in DR (Z = −0.752, *P* = 0.452). Nevertheless, age (*P* = 0.004), BMI (*P* = 0.001), pre-wearing DR (*P* < 0.001) and depths (*P* < 0.001), and changes in depths (Z = −2.236, *P* = 0.025) between the two groups were statistically significant. Patients with smaller initial pressures were more likely to achieve complete correction (*P* = 0.008).

Forty patients reported minor complications, including erythema of the anterior chest wall in thirty-five patients, tension blister in four patients, and transient paresthesia in the left arm in one patient. All of them disappeared in all patients, requiring no special treatment.

### Influencing factors

In the univariable analysis, there were no significant differences between the excellent and non-excellent groups in terms of age, usage time, BMI, sex, symmetry, comorbidity, family history, and thoracic surgery history. In the multivariable analysis, the final models adjusted for initial depths, BMI, treatment periods, initial pressures, symmetry, family history, and complications. The result was that decreased initial depths (OR 0.69, 95% CI 0.58–0.84, *P* < 0.001) and increased treatment periods (OR 1.58, 95% CI 1.23–2.04, *P* < 0.001) were independent predictors of excellent outcomes (Table 2).

**Table 2:** Multivariable logistic regression analysis of the prediction of excellent outcomes based on demographics and VB usage details in patients.

Covariates	OR	95% CI	*P* value
Initial depths	0.69	0.58–0.84	<0.001
BMI	1.04	0.74–1.44	0.839
Treatment periods	1.58	1.23–2.04	<0.001
Initial pressures	0.608	0.86–1.09	0.97
Symmetry	1.06	0.23–4.95	0.945
Family history	0.36	0.12–1.09	0.071
Complications	0.64	0.21–1.94	0.432

## Discussion

This study demonstrated the short-term efficacy of VB in selected preschool PE patients with limited side effects. Patients with decreased initial depths and increased treatment periods are more likely to achieve excellent treatment outcomes, which indicates that intensive treatment may be required in patients with relatively deep depression of the anterior chest wall.

All patients who accepted VB treatment in our study were preschool children. They usually do not meet the surgical indication for the relatively high recurrence rate, although a lack of treatment in this population may result in severe psychological and physical trauma. To explore the effect of VB, a high-resolution 3D scanner was used to monitor the depth changes during follow-up objectively. There is a good correlation between the 3D scanner and tomography scanning results (18). The 3D scanner has avoided radiation-related risks, especially for children, and decreased patient and clinician burdens, representing a desirable measuring method (19).

This study showed a complete correction rate of approximately 30.9% after a median follow-up of nine months, which is comparable to previous observational studies. However, the treatment periods and daily usage time were obviously shorter in this study, perhaps because patients in our study were younger, whose chest wall was still soft, and the depth of chest wall depression was less severe than in previous studies. Our results are in accordance with Lopez, who reported a better correction rate in the pediatric subgroup (11). Obermeyer et al. have also reported better outcomes in children (<18 years) than adults (12). We found statistically significant changes in age and depth of deformity between the two groups according to the initial negative pressure value, which is a more objective reflection of better chest wall flexibility in children. Therefore, the difference in complete correction rate between different age groups might be related to chest wall flexibility. In addition, decreased daily usage time and treatment periods in this study may positively impact the compliance of preschool children.

Contrary to the previous literature, this study showed that age at treatment and symmetry of PE were not statistically significant in a multifactorial logistic regression analysis. Gao et al. analyzed the effects of VB treatment in 82 PE children and showed better outcomes in children up to 10 years of age (14). Obermeyer et al. showed better results in children younger than 11 years of age, possibly because their study included both adult and pediatric patients (12). As the child ages, chest wall stiffness increases and flexibility decreases, thus influencing the outcome. In contrast, our study included all children under the age of seven, and a negative pressure of 15 kPa could elevate 50% of the children's chest to a normal level at the initial outpatient visit. The VB could generate sufficient force to elevate the sunken sternum. Therefore, the impact of age on treatment outcome in this study was limited. In addition, patients with asymmetric PE usually become progressive with age, making it more challenging to keep in place (12). In this study, the degree of deformity in most patients was mild, with 86.3% of them having a symmetrical deformity, so the influence of symmetry on the treatment outcome may not have been found. Another probable reason is that the previous study was retrospective and did not control for potential confounding factors such as BMI and comorbidities, so the results may be biased.

The stratified analysis demonstrated a significant reduction in depths and DR between the two groups according to different usage times, while only depths were different according to different initial pressures. Moreover, although both groups' depths and DR values decreased gradually during treatment, it demonstrated a more notable change in the prolonged daily usage time group, strengthening previous evidence of the recommended instruction of a minimum of 60 min daily (6). Consequently, it may therefore be necessary to closely monitor the use of VB during treatment to improve outcomes.

The effects of daily usage time, initial negative pressure, sex, BMI, symmetry, comorbidities, complications, family history, and history of chest surgery on the treatment outcome were not statistically significant. All patients in this study have no breast development, and sex has a limited effect on treatment outcomes. Therefore, VB treatment at an early age is suitable. Although complications were significantly different in the univariate analysis, they were not significant in the multifactorial model. It is possible that most complications during treatment are minor, such as spotting and erythema, and usually require only brief discontinuation of the VB before regular use can be resumed.

Our study has direct clinical applicability because of the finding of the influencing factors of the treatment outcome. These results that decreased initial depths and increased treatment periods are more likely to achieve excellent treatment outcomes also corroborate the findings of prior work by Toselli et al..(20). As mentioned above, the treatment outcome of VB may be influenced by many factors. Moreover, personalized treatment is urgently needed to find suitable candidates, improve treatment compliance, and improve outcomes. In previous studies, a simple change in the use time, pressure, or frequency of VB during the treatment period in all patients may not address the conflict between compliance and the treatment protocol (14, 15, 21, 22). Although prolonged usage time may be associated with better outcome, it may result in poor treatment compliance and the discontinuation of VB therapy. Our work may provide evidence for personalized treatment in which patients at risk for unsatisfactory treatment may need intensive treatment. In contrast, others may accept regular treatment without additional daily usage times. In addition, our study will enable improved prediction of treatment outcomes at the early stage, which could aid clinicians in being aware of the risk of a failure treatment and in providing proper recommendations.

Our study has several limitations. As mentioned above, VB therapy is a highly personalized treatment modality. It is challenging to record wearing details, although a standardized diary was given to parents to record daily VB usage details. Future electronic VB with sensors that can record time and pressure may solve this problem. Additionally, this study lacks a control group, and patients who refuse to receive both surgery and VB treatment may be included in our future study to confirm VB's effectiveness. In addition, the logistic regression analysis may be confounded by potential factors such as the types or characteristics of the VB. Moreover, the treatment outcome was based on anthropometric measurements, but the quality of life, self-belief changes, and caregivers' think about outcomes were not evaluated. It is essential to include the patients' and caregivers' evaluation of outcomes in future studies. Adapting a questionnaire already developed for pectus carinatum might help improve compliance and treatment outcomes (23). In addition, the likelihood of recurrence in patients who reached normal appearance and the effect of VB on thoracic growth is unclear because of the relatively short follow-up time. Furthermore, studies of the long-term treatment outcomes are necessary.

In conclusion, VB was an effective method with limited side effects, which may be proposed as the first-line treatment in selected PE preschool patients. Factors influencing treatment outcome might include the depth of depression and the duration of treatment, which could aid in developing more personalized treatments in this population. However, further prospective studies are needed to confirm these results.

## Contribution to the field statement

Pectus excavatum is the most common congenital chest wall defect, with an incidence of approximately 1 in 400 to 1 in 1,000 live births. Patients can be exercise intolerance and markedly cosmetic deficiency. And they usually acquired surgical treatment before puberty to reduce growth-related recurrence in patients with early surgery. However, at the time of surgery, some psychosocial issues, such as behavioral disorders, may have already developed at preschool age. This may result in inadequate treatment in preschool patients. The vacuum bell was introduced in 2005 and has been reported as an exciting treatment option in selected patients. The ability to elevate the sternum within several minutes has been approved by intraoperative thoracoscopy, but the excellent correction rate ranges from 13.5% to 43.6% in different studies. However, there are few reports focused on preschool patients. It is unclear the effectiveness of vacuum bell for preschool patients. In addition, studies have suggested that age at treatment, symmetry and initial depth may affect the treatment results. In our study, we demonstrated the effectiveness of vacuum bell in selected preschool patients. We found that decreased initial depths and increased treatment periods might improve the effectiveness, which may provide evidence for personalized treatment.

## Data Availability

The original contributions presented in the study are included in the article/Supplementary Material, further inquiries can be directed to the corresponding author/s.

## References

[1] Fokin AA Steuerwald NM Ahrens WA Allen KE. Anatomical, histologic, and genetic characteristics of congenital chest wall deformities. Semin Thorac Cardiovasc Surg. (2009) 21:44–57. 10.1053/j.semtcvs.2009.03.001 19632563

[2] Sacco-Casamassima MG Goldstein SD Gause CD Karim O Michailidou M Stewart D Minimally invasive repair of pectus excavatum: analyzing contemporary practice in 50 ACS NSQIP-pediatric institutions. Pediatr Surg Int. (2015) 31:493–9. 10.1007/s00383-015-3694-z 25814003

[3] Nuss D Obermeyer RJ Kelly RE. Pectus excavatum from a pediatric surgeon's Perspective. Ann Cardiothorac Surg. (2016) 5:493–500. 10.21037/acs.2016.06.04 27747183 PMC5056929

[4] Ji Y Liu W Chen S Xu B Tang Y Wang X Assessment of psychosocial functioning and its risk factors in children with pectus excavatum. Health Qual Life Outcomes. (2011) 9:28. 10.1186/1477-7525-9-28 21542911 PMC3098203

[5] Eisinger RS Islam S. Caring for people with untreated pectus Excavatum: an international online survey. Chest. (2020) 157:590–4. 10.1016/j.chest.2019.10.034 31730833

[6] Schier F Bahr M Klobe E. The vacuum chest wall lifter: an innovative, nonsurgical addition to the management of pectus excavatum. J Pediatr Surg. (2005) 40:496–500. 10.1016/j.jpedsurg.2004.11.033 15793724

[7] Haecker F-M Sesia S. Vacuum bell therapy. Ann Cardiothorac Surg. (2016) 5:440–9. 10.21037/acs.2016.06.06 27747177 PMC5056932

[8] Patel AJ Hunt I. Is vacuum bell therapy effective in the correction of pectus excavatum? Interact Cardiovasc Thorac Surg. (2019) 29:287–90. 10.1093/icvts/ivz082 30919892

[9] Stagnaro N Trocchio G Torre M Rizzo F Martucciello G Marasini M. Cardiovascular MRI assessment of pectus excavatum in pediatric patients and postoperative simulation using vacuum bell. J Pediatr Surg. (2021) 56:1600–5. 10.1016/j.jpedsurg.2020.11.017 33256973

[10] Haecker F-M Sesia SB. Intraoperative use of the vacuum bell for elevating the sternum during the Nuss procedure. J Laparoendosc Adv Surg Tech A. (2012) 22:934–6. 10.1089/lap.2012.0030 23137116

[11] Lopez M Patoir A Costes F Varlet F Barthelemy J-C Tiffet O. Preliminary study of efficacy of cup suction in the correction of typical pectus excavatum. J Pediatr Surg. (2016) 51:183–7. 10.1016/j.jpedsurg.2015.10.003 26526206

[12] Obermeyer RJ Cohen NS Kelly RE Ann Kuhn M Frantz FW McGuire MM Nonoperative management of pectus excavatum with vacuum bell therapy: a single center study. J Pediatr Surg. (2018) 53:1221–5. 10.1016/j.jpedsurg.2018.02.088 29606411

[13] St-Louis E Miao J Emil S Baird R Bettolli M Montpetit K Vacuum bell treatment of pectus excavatum: an early North American experience. J Pediatr Surg. (2019) 54:194–9. 10.1016/j.jpedsurg.2018.10.011 30414687

[14] Gao Y Li J-H Yu J-G Tan Z Liang L Huang T Noninvasive treatment of pectus excavatum with a vacuum bell combined with a three-dimensional scanner. Pediatr Surg Int. (2020) 36:1205–11. 10.1007/s00383-020-04726-9 32789545

[15] Deng X Huang P Luo J Wang J Yi L Yang G A novel three-dimensional printed vacuum bell for pectus excavatum treatment: a preliminary study. J Cardiothorac Surg. (2020) 15:240. 10.1186/s13019-020-01276-y 32912269 PMC7488022

[16] Alaca N Alaca I Yüksel M. Physiotherapy in addition to vacuum bell therapy in patients with pectus excavatum. Interact Cardiovasc Thorac Surg. (2020) 31:650–6. 10.1093/icvts/ivaa161 32960955

[17] Deng X Huang P Luo J Wang J Yi L Yang G The consistency of an optical body surface scanning method compared with computed tomography: a validation study. J Pediatr Surg. (2020) 55:1448–52. 10.1016/j.jpedsurg.2019.07.015 31455544

[18] Hussain A Patel A Hunt I. Are non-radiation-based imaging modalities effective for objectively assessing and monitoring patients with pectus deformities? Interact Cardiovasc Thorac Surg. (2020) 31:536–9. 10.1093/icvts/ivaa134 32964931

[19] Fuentes S Pradillos-Serna JM Berlioz M Damián-Salamanca F Lorenzo T Ardela-Díaz E. Validating 3D indexes in the non-surgical pectus excavatum patient. J Pediatr Surg. (2021) 56:230–4. 10.1016/j.jpedsurg.2020.06.006 32650999

[20] Toselli L Chinni E Nazar-Peirano M Vallee M Sanjurjo D Martinez J Determinants of success associated with vacuum bell treatment of pectus excavatum. J Pediatr Surg. (2022). 10.1016/j.jpedsurg.2022.04.010 35525808

[21] Toselli L Vallee M Elmo G Martinez J Sanjurjo D Nazar M Implementation and acceptance rates of a specially designed vacuometer for the vacuum bell treatment of pectus excavatum. J Pediatr Surg. (2021) 56:2235–8. 10.1016/j.jpedsurg.2021.03.008 33789800

[22] Furuta S Nagae H Ohyama K Tanaka K Kitagawa H. The vacuum treatment for the pectus excavatum thickened subcutaneous fat of the chest wall and is effective in preteenagers. Pediatr Surg Int. (2020) 36:1465–9. 10.1007/s00383-020-04758-1 33125551

[23] Pessanha I Severo M Correia-Pinto J Estevão-Costa J Henriques-Coelho T. Pectus Carinatum evaluation questionnaire (PCEQ): a novel tool to improve the follow-up in patients treated with brace compression. Eur J Cardiothorac Surg. (2016) 49:877–82. 10.1093/ejcts/ezv198 26059874

